# Glycerol Droplet Spreading on Growing Bacillus Subtilis Biofilms

**DOI:** 10.3390/mi14030599

**Published:** 2023-03-04

**Authors:** Siyang Luo, Yanan Liu, Hao Luo, Guangyin Jing

**Affiliations:** School of Physics, Northwest University, Xi’an 710127, China

**Keywords:** spreading, wetting, biofilms

## Abstract

Bacterial biofilm is a three-dimensional matrix composed of a large number of living bacterial individuals. The strong bio-interaction between the bacteria and its self-secreted matrix environment strengthens the mechanical integrity of the biofilm and the sustainable resistance of bacteria to antibiotics. As a soft surface, the biofilm is expected to present different dynamical wetting behavior in response to shear stress, which is, however, less known. Here, the spreading of liquid droplet on *Bacillus subtilis* biofilm at its different growing phases was experimentally investigated. Due to the viscoelastic response of the biofilm to fast spreading of the droplet, three stages were identified as inertial, viscous stages, and a longer transition in between. The physical heterogeneity of growing biofilm correlates with the spreading scaling within the inertial stage, followed by the possible chemical variation after a critical growing time. By using the duration of inertial spreading, the characteristic time scale was successfully linked to the shear modulus of the elastic dissipation of the biofilm. This measurement suggests a facile, non-destructive and in vivo method to understand the mechanical instability of this living matter.

## 1. Introduction

Bacteria attach to wet surfaces and then gradually grow into a multicellular and surface-adherent community to form the so-called bacterial biofilm, which has been recognized as the main cause of infection diseases, and even worse with sustainable antibiotic resistance [1,2,3]. Bacterial films can also affect ecology by altering soil wettability [4]. This type of special film is not just made from aggregation of bacteria, but a living three-dimensional matrix composed of self-secreted extracellular polymeric substances able to defend against various drugs and environmental pressure, and to host the immune system [5,6]. This self-produced matrix possesses mechanical stability and acts as the scaffold of the biofilm [7]. Chemical agents and biocides delivered in liquid form are used for treatment against the microbes inside the biofilm, i.e penetration of drug suspension into the biofilm interior. Intuitively, hydrophobicity and oleophobicity of biofilms might play a possible role in repelling the drug solution, which acts as a link to drug resistance [8]. The wettability of biofilms is very important for the formation of bacterial communities, and it can also be used as a parameter to characterize the state of biofilms on a macroscopic scale [9]. Therefore, the wetting properties of the biofilm suggest a practical opportunity for biocides to reach target cells enclosed in the biofilm. Proteins and biopolymers self-secreted by the bacteria were identified to be water-repellent and gas-resistant, responsible for extreme hydrophoboicity and oleophobicity [10,11,12,13].

Based on the traditional methods dealing with wetting properties of classical solid surfaces, the interfacial morphology and chemical composition of the biofilms are recognized as the dominant factors relevant to biofilm wettability. However, the studies on static wettability are not adequate to characterize the interaction between biofilms and liquids. Biofilm is a soft matrix with *living* components from dynamical secretion, and even its viscoelastic response makes the wetting behavior different from that on normal solid and soft materials [14,15]. The dynamic interaction between the liquid or flows and the biofilm surface is primarily important, particularly when soluble drugs come into contact with the exterior of the biofilm. While the dynamics of liquid droplet spreading on solid surfaces have been studied extensively [16,17], and on soft surfaces as well [18,19,20,21], dynamical wetting on biofilms remains unknown. The dynamic spreading of a sessile droplet on biofilm is expected to exhibit distinct behavior, wherein the liquid droplet deforms the biofilm close to the contact line by surface tension.

For this purpose, in the present work, spreading of glycerol droplet on the biofilm of *Bacillus subtilis* (shorten as B. subtilis) was employed to quantitatively investigate the interaction between the droplet in motion and the soft biofilm. By using a fast camera, the short dynamics of spreading of the droplet on the biofilm was obtained, allowing us to illustrate the scaling law compared to the one on traditional passive counterparts. More interestingly, the growing biofilm in its life cycle was directly used for the measurement, showing a strong correlation between its growing stage and the spreading law. By including the viscoelasticity of the biofilm, the shear modulus was quantitatively determined from the characteristic time of spreading dynamics. Application of the fast spreading dynamics of a droplet on bacterial biofilm not only mimics the spreading kinetics of pharmaceutical suspension on such special soft membrane, but also infers desirable information, such as viscoelasticity and stiffness of biofilm, in understanding the mechanical properties of bacterial biofilm. Such information is, however, difficult to access by traditional measurement. This mechanical response of biofilm offers the practical possibility to understand the mechanical integrity of biofilm in its growing state in a non-destructive and real-time way.

## 2. Materials and Methods

*B. subtilis* (strain BS168) was used here to form biofilm on an agar gel plate. The bacteria were cultured using a standard protocol. In brief, the bacteria were taken out from a frozen tube stored at $-80{\;}^{\circ }$C, and then transferred into Luria–Bertani broth (LB) medium for overnight growth at $30{\;}^{\circ }$C on a stage shaking at the speed of 200 rpm. The culture medium LB was made before the experiment from the components of tryptone 1.0%, yeast extract 0.5%, and NaCl 1.0%. Note that the concentrations of the agents were all in the unit of weight by volume percent (*w*/*v* %). Thereafter, a controlled volume of this bacterial suspension was dispersed into the new LB medium with the resultant concentration of optical density OD ≈0.05 measured under the optical wavelength of 600 nm. Then, the bacterial suspension in this fresh LB medium was transfered on to shaking stage for another growing process at $30{\;}^{\circ }$C on the same shaker. After 6 hours, the bacteria arrived the mid-exponential phase of growth. A small amount of this final bacterial solution (with OD = 0.5) was gently spread onto the agar plates in petri dishes. These petri dishes were then sealed and placed upside down in an oven at $30{\;}^{\circ }$C. Every 12 h, a set of gel-like blocks were cut out from each agar plate for further experiments. Usually, for reproducible measurement, at least 5 identical blocks from the same agar plate were picked up at its certain growth time.

**Figure 1:**
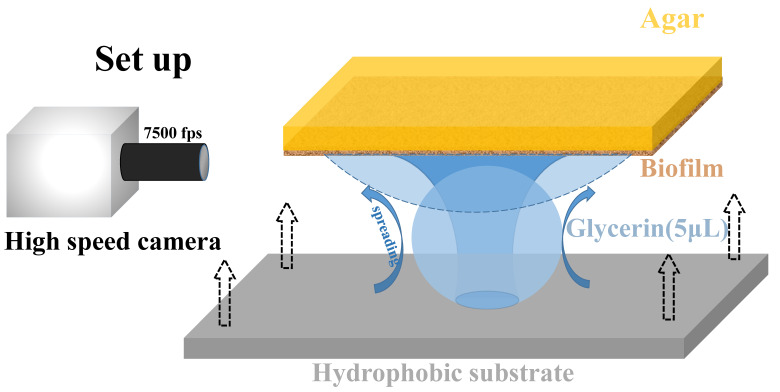
Experimental setup of glycerol droplet spreading on biofilm. A glycerol droplet with a volume of ∼5 μL (radius $R_{0}∼$1 mm) was initially introduced onto a superhydrophobic glass slide below. The upside down biofilm, with different growing times, gently approached the sessile droplet on the substrate beneath. A fast camera aside was used to record the whole spreading process and the detachment of the droplet from the lower substrate, at a frame rate of 7500 fps.

Droplets were produced from glycerol solution (99%, Fuyu Chemicals, Tianjing, used as received), with a volume about 5 μL, and initial radius of $R_{0}∼1\;$mm. In order to capture the spreading process, a high-speed camera (Phantom, VE0710L), equipped with objective AF Micro Nikkor (60 mm f/2.8D) was used. Glass slides with superhydrophobic substrate were prepared before the droplet spreading experiments. The superhydrophobic substrates were obtained by baking Glaco (Mirror Coat Zero, SOFT99, Japan) onto the cover glass 3 times. During the spreading process, in order to limit the initial velocity and the vibration from the impact of the droplet on the substrate, the upside down biofilm was carefully moved towards the droplets sitting on the superhydrophobic substrate underneath (Figure 1). The biofilm sample approached the droplet gently from above until the biofilm surface just touched the glycerol droplet, and then recording process was started immediately by the fast camera. Since the contact angle of glycerin droplet on the superhydrophobic glass slide could reach 160∼170°, the droplet was easily detached from the substrate due to limited contact area on the substrate. The 3D profiles of the biofilms were taken under the optical profiler of a white-light interference microscope (Talysurf CCI 6000). The contact angle was measured by the home-made imaging setup, for which a camera ( AVT F131B CCD ) was used to take snapshots from side view, followed by the contact angle measurement with Matlab coding.

## 3. Results and Discussion

### 3.1. Fast Spreading of Droplet on Growing Biofilm

During the very early stage of the droplet spreading, the surface tension converted the excess free energy to drop motion and accelerated the liquid layer into rapid expansion. The radius R(t) of the contact base of the droplet increased with time as a power law $R∼t^{\alpha }$, with the scaling factor α = 1/2 [22]. Thereafter, the droplet spreading slowed down significantly, due to the viscous dissipation called viscous regime, described by the famous Tanner’s law R∼$t^{1/10}$, which has been widely confirmed [23,24,25]. Distinct from a traditional solid substrate, here, biofilms at different growing stages were soft, and the living biofilms were dynamically evolving with time (usually in hours). Note that, however, the glycerol droplet spreading on the biofilm occurred at a time scale of microseconds, which showed the short dynamical behavior.

**Figure 2:**
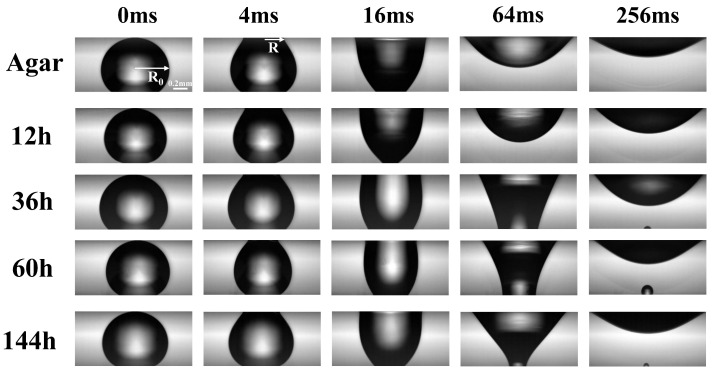
Snapshots of droplet shapes during the spreading. Left to right: snapshot at different spreading times $t_{s}$ in microseconds. Top to bottom: droplets on biofilm samples with different growing times $t_{g}$ in hours.

Since the surface energy of the upper surface (biofilm, hydrophilic), was higher than that of the one below (superhydrophobic glass), thus the droplet was easily transferred onto the biofilm from the glass slide below and immediately expanded onto the biofilm. Figure 2 shows the sequential snapshots over time $t_{s}$ of the spreading processes on the different biofilms growing with time duration of $t_{g}$ (in hours) from the beginning of culturing on agar plates. Here, the time zero was set as the moment of the droplet immediately touching the biofilm at its apex. Vertically, in Figure 2, droplet shapes on different biofilms picked up at different growing times $t_{g}$, are compared at the same moment of spreading time. The contact area of glycerol with the hydrophobic substrate gradually decreased during the spreading of glycerol droplet on the bacterial film, as shown in the snapshot at 256ms (Figure 2). All of the droplets were able to spread onto the biofilms driven by surface tension and, finally, stuck onto the biofilms with a pinned contact line after ∼250 ms.

**Figure 3:**
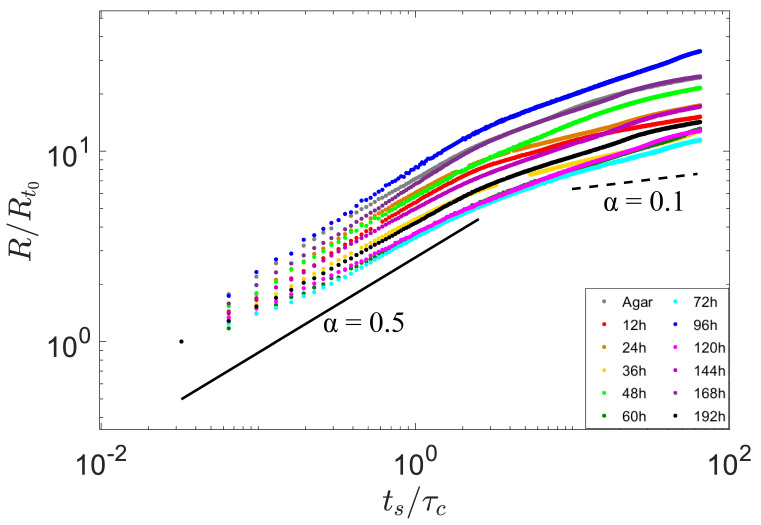
Scaling laws of spreading scaling. The normalized spreading radius *R* as $\left(R/R_{0}\right)$ was dependent on normalized time $t_{s}$ as $t_{s}/\tau_{c}$, where $R_{0}$ was the initial radius of each droplet, and $\tau_{c}$ was the characteristic inertial time scale(($\rho R_{0}^{3}{/\gamma )}^{1/2}$, the initial droplet size ($R_{0}$), the density (ρ), and the surface tension (γ)).

The contact radius $R(t_{s})$ between the droplet and the biofilm increased over time, which was normalized as $R/R_{0}$ and plotted in Figure 3. The spreading process can be quantitatively expressed as a scaling relation of $R/R_{0}∼t_{s}^{\alpha }$ with power exponent α, where $R_{0}$ is the initial radius of each droplet.

**Figure 4:**
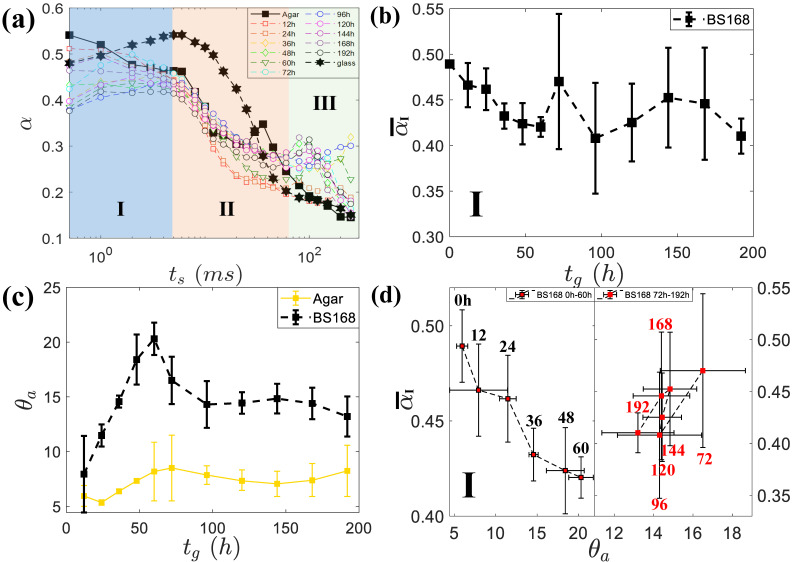
Exponent α dependent on the spreading time $t_{s}$, and the mean value ${\bar{\alpha }}_{I}$ in the inertial stage dependent on the growing time of biofilm and its wettability. (**a**). The spreading exponent α varied with time, distinguishing three stages: inertial stage (I), transition stage (II), and viscous stage (III). (**b**). The spreading exponent averaged on repeated measurements, ${\bar{\alpha }}_{I}$ in the inertial phase, depending on the growing time of the biofilm. (**c**). Advancing contact angle $\theta_{a}$, depending on the biofilm with increasing growth time. The yellow points are the control measurement for the bare agar gels. (**d**). ${\bar{\alpha }}_{I}$ varied with the advancing contact angle $\theta_{a}$.

Biofilm is recognized as a type of soft material composed of living matter secreted by individual bacteria during their growth. Thus, excessively, a droplet spread on biofilm shows viscoelastic response to the driving force from the surface tension at the droplet edge. This viscoelastic nature of soft biofilm significantly changes the dynamics of droplet spreading, i.e., regulating the relaxation process in response to force exerted by the surface tension at the contact line of the droplet. Here, the entire spreading process is divided into three stages: I, inertial stage; II, transition stage; and III, viscous stage. Stage I represents the initial spreading, in which fast moving of the droplet contact line on the biofilm surface is allowed. During this stage, the typical speed of the moving droplet contact line can approach ∼1 mm /s driven by the surface tension, and has exhibits a spreading exponent close to α∼ 0.5 (blue region in Figure 4a). In stage II, a transition from inertial to viscoelastic occurs, and lasts over tens seconds (orange region in Figure 4a). Thereafter, the droplet gradually comes to rest and forms an equilibrium shape with a static contact angle, due to the balance between the viscous friction and the driving force of surface tension. Considering the classic scaling law $R∼v^{3/10}{\left(\gamma t/\eta \right)}^{1/10}$, with droplet volume v, viscosity η, density ρ, and surface tension γ [26], the final spreading exponent is close to 0.1 (light green region in Figure 4a). Note that the exponent α measured on some biofilms at certain growing times $t_{g}$, was dramatically scattered in stage III. The scattering here was due to droplet vibration on biofilms, resulting from the capillary wave generated when the droplet just detached from the substrate below.

It is interesting to correlate the spreading exponent ${\bar{\alpha }}_{I}$ at the stage I average on repeated measurements to the growth time $t_{g}$ of different biofilm phases, as shown in Figure 4b. It can be seen that ${\bar{\alpha }}_{I}$ decreases monotonically with growth time $t_{g}$ within the duration of 0∼60h, while the data from 60 ∼192h has no obvious trend but possesses a large standard deviation.

The question is how to understand the dependence of the exponent α on growth $t_{g}$ time? After the seminal work on short dynamics of spreading on hydrophilic surfaces with α=0.5 [22], the smaller value of this exponent was nicely determined for spreading on surfaces of partial wetting [27]. These results remind us that biofilm at different growing phases should possess different wetting properties. Indeed, biofilms at different phenotypic exhibit varying interfacial morphologies [28], and even adaptively generate different chemical components, as well, which can jointly affect the static contact angle. Due to the fact that the glycerol droplets prefer to wet the biofilm, the advancing contact angle $\theta_{a}$ is a good measurement to characterize the static wetting property. Obviously, contact angle $\theta_{a}$ increases with growth time $t_{g}$, and reaches a plateau after about 60 h (Figure 4c). In order to remove the influnce induced by the supporting agar gel underneath, corresponding control experiments on agar surfaces were performed, as shown in the yellow curves in Figure 4c. Then, the dependence of ${\bar{\alpha }}_{I}$ on θ${}_{a}$ for biofilms at different growth times was plotted in Figure 4d. During the early growth time 0∼60h, ${\bar{\alpha }}_{I}$ linearly decreases with $\theta_{a}$, which is in a good agreement to the findings on traditional solid substrate with varying wettability [27]. At the later growth time $t_{g}$ (after 60 h), the biofilm tends to be mature and shows a slightly smaller contact angle, and possesses a scattering of spreading exponent ${\bar{\alpha }}_{I}$ due to possible additional chemicals secreted from individual bacteria under starvation.

**Figure 5:**
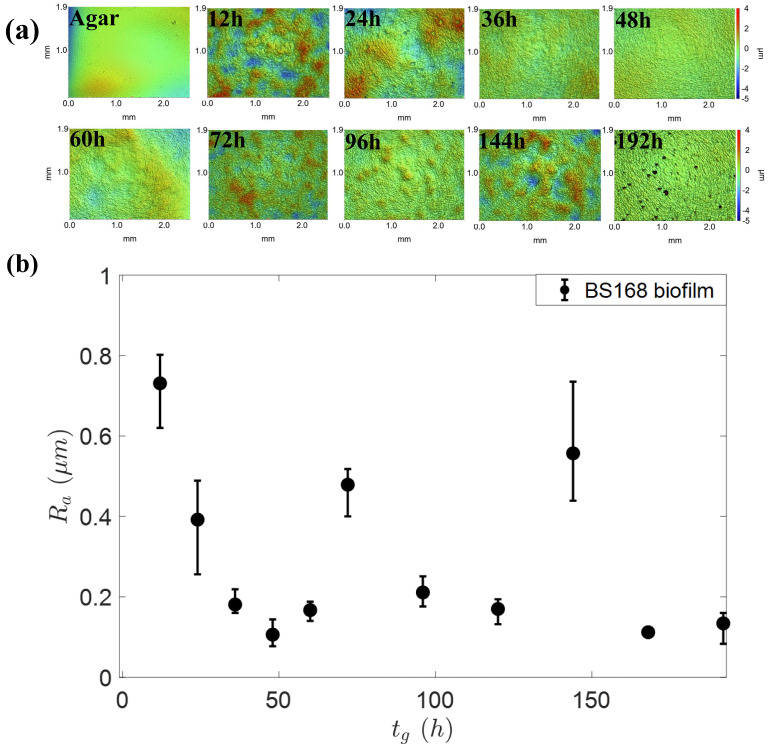
Surface topography of biofilm in different growth time. (**a**) The 3D profiles of biofilm surfaces under optical profilometer with 2.5× objective lens; (**b**) Mean roughness $R_{a}$ vs. growth time $t_{g}$.

Then, the questions also arises as to how and why the contact angle changes with the growth time of the biofilm? The wetting properties of a surface can be tuned by the surface features (physical heterogeneity), and chemical inhomogeneity. The influences of these surface properties on wettability are mostly coupled together, making the analysis very complicated [29,30]. Therefore, a common method is to find the main parameters from various influencing parameters according to data fitting. It was reported that the topography of the biofilm surface (bacteria *Bacillus subtilis*) changed its wettability in different growth phases [12,31]. Here, the roughness of the biofilm surface during continuous growth is characterized by using a white-light interferometric profilometer. Figure 5a shows the topography of the biofilm at different growth times. It can be seen that the biofilm surface becames smoother before 60 h, and then gradually reaches saturation. The average surface roughness $R_{a}$ depended on the growth time, as shown in Figure 5b. Additionally, the agar was very smooth, and its $R_{a}$ was just 0.057 ± 0.03 μm.

Since the biofilms are in a glycerin-friendly state (the contact angle was less than 20°), the glycerol droplets are expected to be in the Wenzel state, i.e., the contact angle depends on Young’s contact angle θ${}_{Y}$ and roughness *r* as $\cos\theta_{w}=r\cos\theta_{Y}$ [29]. The Wenzel model predicts that the surface roughness *r* is able to amplify the hydrophilicity, i.e., smoothing of the biofilm increases the contact angle. It is consistent with the measurements here that biofilm becames smooth with growth time in the range of 0∼60h, and, consequently, the contact angle increases correspondingly, shown in Figure 4c. Therefore, we believe that roughness is the main parameter to regulate the wettability of bacterial film at this formation stage. In this stage, as the contact angle increases, the $\alpha_{I}$ of the inertia stage decreased monotonically (as shown in Figure 4d). We are pleased that this trend was consistent with the data of Nita et al. [30] and Bird et al. [32] However, when the growth time was longer than 60 h, both the roughness and the contact angle data are largely scattered, reminding us that chemical heterogeneity may play a role. The bacteria can completely cover the agar surface in about 12h to form biofilm composed of densely-packed bacteria. It has been reported that once the biofilm formed, the individual bacteria inside the biofilm started to secrete mucus (polymer matrix) to promote biofilm expansion [33]. More interestingly, it was confirmed that biofilm of the *Serratia* bacteria species secreted chemicals with different concentrations at different growth stages [28], which might explain, here, the saturation but scattered roughness and spreading exponents after 60 h.

### 3.2. Viscoelastic Response of Biofilm to the Droplet Spreading

**Figure 6:**
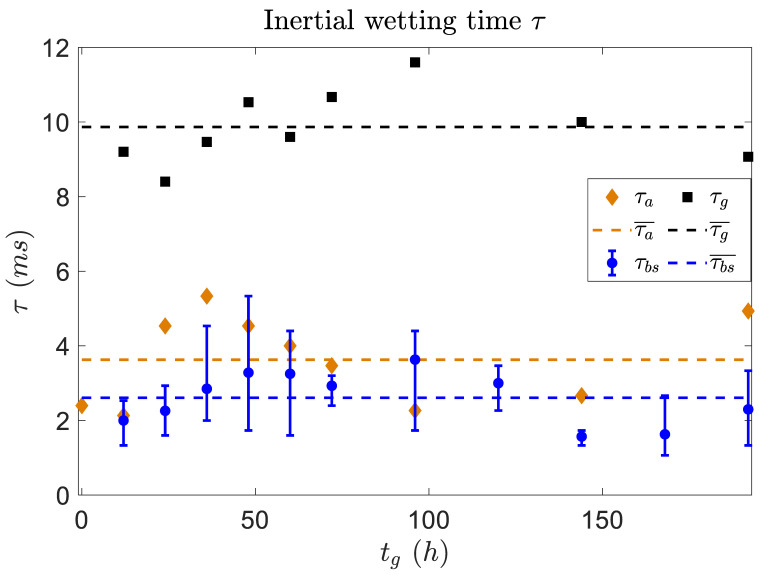
Characteristic time scales τ of inertial spreading. Values of τ are extracted from Figure 4, correspondingly for droplets spreading on biofilm (blue markers), bare agar gel (orange markers), and a normal glass slide (black squares) as control experiment.

Biofilm is composed of relatively rigid bacteria but also a very soft polymer matrix secreted by the bacteria themselves. Therefore, biofilm is expected to show viscoelastic response and dynamic deformation under the exerted surface tension during droplet spreading. It was found by Chen et al. that the power law for the early inertial stage on a soft substrate is mainly dependent on the substrate’s wettability rather than its softness [34]. In this report, the duration time τ of fast inertial spreading was however confirmed to be affected by the softness of the soft substrate. After this report, Chen et al. also presented a quantitative model to capture the transition from rapid inertial spreading to slower viscoelastic spreading [35]. This duration of the inertial state τ can characterize the contributions from both the softness and hardness of the biofilm. As shown in Figure 6 in our cases, the mean inertial time ${\bar{\tau }}_{g}$ by averaging on repeated measurements of glycerol droplets spreading on glass (typical hard substrate) was determined as about 10 ms, while about 3.6 ms on typical soft agar gels used in our experiments. Further, ${\bar{\tau }}_{g}∼2.6$ ms of glycerol droplets on our biofilm is also obtained with a value slightly lower than that on bare agar gel.

The accuracy and consistency of our measurements on the persistent time τ of inertial spreading are checked. Firstly, the dynamic spreading of the glycerol droplet on glass is different from that of a pure water droplet. A water droplet spreading on glass exhibits a cross-over time at the moment when the spreading radius is equal in the inertial and viscous regime as: [22]

(1) $$\tau ∼\left(\rho R_{0}\gamma /\eta^{2}\right){\left(\rho R_{0}^{3}/\gamma \right)}^{1/2}$$

This equation above approximately determines the time scale of inertial duration, depending on the initial droplet size ($R_{0}$), liquid viscosity (η), density (ρ), and surface tension (γ). Note that the glycerol droplet adsorbs water moisture (∼46%*w*/*v*) easily, which in our experiment has the resultant properties of ρ≈1137 kg/m${}^{3}$ and γ≈67.2 mN/m. Furthermore, due to the heat effect from the light power used by the fast camera, the viscosity of the glycerol droplet was estimated as η=5.4 mPa·s [36]. Therefore, the calculated τ was about 10 ms, which is highly consistent with our measured value (Figure 6).

In regard to spreading on agar gel, the inertial time τ mainly depended on the surface tension and the hardness of the substrate. In the preparation of agar gel from its powder, the agar content controls the hardness of the agar surface in gel form [37,38]. The mean inertial wetting time ${\bar{\tau }}_{a}$ on agar was about 3.6 ms, which gave the shear modulus of |G≈23 kPa by using the data in Ref. [34] for normal soft surfaces, and is consistent with the typical value of shear modulus of agar gel [39]. Similar to the passive soft surface, biofilm can also deform out-of-plane under the vertical component of surface tension at the line of contact. The shear modulus |G| of biofilm plays an important role during droplet spreading, during which the biofilm surface is pulled up and deformed to form a *ridge*. In the early stage of spreading, the energy dissipation restored into the deformation of the biofilm is much larger than that of viscous dissipation, and, thus, the viscosity effect at this stage can be ignored [40]. The viscoelastic dissipation process during the droplet spreading can be fitted by the model in [35] as:

$$r\approx \xi \left(1-e^{-(\xi /\Psi )t}\right)=R_{0}{\left(2\left(1+\cos\theta_{eq}\right)\right)}^{1/2}\left[1-e^{-(\frac{{\left(2\left(1+\cos\theta_{\mathrm{eq}}\right)\right)}^{1/2}G\epsilon^{2}}{\gamma \varphi \tau R_{0}})t}\right]$$

where $\theta_{\mathrm{eq}}$ is the equilibrium contact angle, and $\Psi =R_{0}^{2}\gamma \omega \tau /2G\epsilon^{2}$, $\xi^{2}=2R_{0}^{2}\left(1+\cos\theta_{eq}\right)$ with coefficient C. The relaxed energy $G_{r}$ (viscous contribution), and unrelaxed energy $G_{u}$ (elastic contribution), jointly determine the viscoelastic response from a soft substrate. By fitting the data with the parameter $\varphi ={\left(G_{r}/G_{u}\right)}^{1/2}-{\left(G_{r}/G_{u}\right)}^{-1/2}=\sqrt{\beta }-\sqrt{1/\beta }$ in our case, the mean value of φ≈0.6 is obtained, which is nicely consistent with the result (φ=0.3) for normal soft surface in Ref. [35]. As a consequence, the inertial time τ on biofilm is derived from the direct measurement with the droplet spreading in the short dynamical regime, i.e., the inertial stage of spreading.

From the discussion above it is convincing that the hardness of the soft surface can be estimated from the measurement of inertial time τ. Thus, it is intriguing to evaluate the hardness of the biofilm. Since there is no report on the inertial time τ on biofilm, its comparison to the counterpart of soft surfaces is demanding. The inertial time on biofilm, here, had an average value of about $\bar{\tau }\approx$ 2.6 ms, by which the shear modulus |G|≈11 kPa could be determined from the data table in Ref. [34], which is in agreement with the measured value $10^{2}$ Pa to $23\times 10^{3}$ Pa in Ref. [41]. The shear modulus of the biofilm also showed a scattering within a reasonable range.

## 4. Conclusions

In this work, we experimentally investigated the spreading behavior of a glycerol droplet on *B. subtilis* biofilm at its different growing times, and proposed the mechanical analysis on the viscoelastic response from the living biofilm to the surface tension. The results show that the spreading radius exhibits a power law over time, allowing us to analyze the spreading scalings exponent, which defines the inertial stage at a very short time, the followed longer transitional stage, and the last viscous stage. It is found that this specific bacterial biofilm continuously grows with from individual bacteria on nutrient-rich gel plate, exhibiting a smoothening process reflected by biofilm roughness over time in the early stage of growth (12–60 h). There is a critical moment $t_{g}^{*}∼60\;$ h, which separates the dominant factor of the physical heterogeneity of the biofilm responsible for the increasing contact angle. Later, the contribution by the chemical component of the bacterial secretions dominates. This roughness variation contributs to the partial wettability of the biofilm, i.e., increases the static contact angle of the glycerol droplet, and, therefore, extends the duration time of inertial regime. Intriguingly, the viscoelastic response of the biofilm, similar to that of a soft surface, plays an important role in the spreading dynamics, particularly in the inertial stage at the beginning of spreading. Elastic and viscous components compete with each other during the inertial stage, and determine the persistent time of the inertial stage. By using this time scale of inertial duration, the shear modulus of the biofilm is obtained with a reasonable value of G≈11 kPa. The viscoelastic properties might reflect the biofilm adaptation on surfaces and adaptive response to different shear environments. Thus, appropriate characterizations on properties of biofilms can help us to elucidate the role of biofilm mechanics for sessile survival in flowing environments. Our mechanical analysis, based on droplet spreading, could offer the mechanical characterization on living and soft biofilms. It is worth noting that the diversity and dynamic evolution of living materials consisting of microorganisms ensure variability in response to mechanical cues, i.e., surface tension. Here, biofilm from one specific microbe (*B. Subtilis*) is used as the model system to demonstrate the wetting properties and viscoelasticity characterization of living matter, so that the complication result from particularly biofilm with dynamic and complex structures should be considered. To say, biofilm with other strains of bacteria, or growing on different substrates, could vary to some extent.

## Data Availability

Not available.
